# Complete Genome Assembly of a Multidrug-Resistant New Delhi Metallo-β-Lactamase 1 (NDM-1)-Producing Escherichia coli Human Isolate from a New Zealand Hospital

**DOI:** 10.1128/MRA.00780-20

**Published:** 2020-08-20

**Authors:** Catrina Olivera, Jasna Rakonjac

**Affiliations:** a School of Fundamental Sciences, Massey University, Palmerston North, New Zealand; Loyola University Chicago

## Abstract

We report the complete genome of a multidrug-resistant Escherichia coli strain isolated from a New Zealand patient with a history of hospitalization in India. The strain, carrying eight plasmids, harbors chromosome-encoded *nfsA* and *nfsB* mutations, which cause nitrofuran resistance, and class C β-lactamase (*bla*_EC_) and plasmid-encoded *bla*_NDM-1_, *bla*_CTX-M-15_, and *bla*_CMY-6_, as well as other antibiotic resistance genes.

## ANNOUNCEMENT

Escherichia coli NZRM4457 (originally ARL09/232) was the first New Delhi metallo-β-lactamase (NDM)-producing *Enterobacteriaceae* strain to be reported in New Zealand (1). The strain was isolated in 2009 from a urine sample from a patient who had been hospitalized in India and was repatriated to a New Zealand hospital 2 months later.

The E. coli NZRM4457 strain was obtained from the New Zealand Reference Culture Collection (Institute of Environmental Science and Research, New Zealand). We used a Qiagen UltraClean microbial DNA kit to extract genomic DNA from an overnight culture grown at 37°C in LB medium. The DNA sample was then submitted to the Massey Genome Service (Massey University, Palmerston North, New Zealand) for Illumina Nextera XT DNA library preparation and 2 × 150-base paired-end sequencing on the Illumina MiSeq platform. The resulting 3,261,556 reads (90× coverage) were trimmed of adapters using fastq-mcf v1.04.636 in ea-utils (2). Low-quality bases (Q scores of <30) were trimmed and short reads (<25 bp) were removed using SolexaQA++ v3.1.7.1 (3).

The long-read sequencing library was prepared from 1 μg DNA using an Oxford Nanopore Technologies (ONT) ligation sequencing kit (SQK-LSK109) according to the manufacturer’s instructions. The sample was loaded onto a FLO-MIN106 R9.4.1 flow cell and sequenced on the MinION Mk1B platform, yielding 668,000 reads (5.2 Gbp [977× coverage]; mean read length, 5,794 bp). The reads were base called using ONT Guppy v3.4.5, and then the adapters were trimmed with Porechop v0.2.4 (https://github.com/rrwick/Porechop). FiltLong v0.2.0 (https://github.com/rrwick/Filtlong) was used to filter the reads by length (>1,000 bp) and by quality (mean base Q score of >10) and to discard the worst 10% of reads. The reads were randomly subsampled down to 500,003,523 bp (93× coverage) using seqtk v1.3-r106 (https://github.com/lh3/seqtk).

We used the Unicycler v0.4.8 pipeline (4) for hybrid assembly in normal mode, which resulted in nine circular contigs totaling 5,322,305 bp. The assembly was evaluated using Quast v5.0.2 (5), and genome completeness was assessed using BUSCO v4.0.5 (6) and CheckM v1.1.2 (7); 100% of conserved BUSCO genes in the enterobacterales_odb10 data set were recovered from the assembly, and the assembly was 99% complete according to CheckM using a taxonomy-specific workflow for Escherichia coli. The genome was then annotated using the NCBI Prokaryotic Genome Annotation Pipeline v4.11 (8).

The E. coli NZRM4457 genome consists of one chromosome and eight plasmids, which are described in Table 1, with 5,125 coding sequences, 89 tRNAs, 22 rRNAs, and 10 noncoding RNAs. The plasmids were assigned to replicon types using PlasmidFinder v2.0.1 (9), and antimicrobial resistance genes were identified *in silico* using NCBI AMRFinder (10) and ResFinder v3.2 (11) (Table 1). AMRFinder and ResFinder identified genes and mutations that confer resistance to β-lactams, fluoroquinolones, aminoglycosides, bleomycins, and sulfonamides. Additionally, chromosome-borne *nfsA* and *nfsB* were annotated as pseudogenes in E. coli NZRM4457, having internal nonsense (stop codon) and frameshift mutations, respectively. Mutations in *nfsA* and *nfsB* are implicated in nitrofuran resistance (12, 13), and we have verified the strain as being nitrofuran resistant (nitrofurantoin MIC, 128 μg/ml; furazolidone MIC, >128 μg/ml) using the broth microdilution method, according to Clinical and Laboratory Standards Institute (CLSI) guidelines (14).

**TABLE 1:** Characteristics of the genome of Escherichia coli NZRM4457

Component	Size (bp)	G+C content (%)	Plasmid replicon type(s)	Antimicrobial resistance gene(s)
Chromosome	4,836,724	50.9		*bla*_EC_, *nfsA* mutation, *nfsB* mutation, *gyrA* (S83L, D87N), *parC* (S80I), *parE* (S458T)
Plasmid 1	230,339	47.3	IncHI1A, IncHI1B	*bla*_TEM-1_, *bla*_NDM-1_, *bla*_CTX-M-15_, *ble*
Plasmid 2	139,294	51.2	IncC	*bla*_CMY-6_, *aac(6′)-Ib3*, *qacEdelta1*, *aph(3′)-Ia*, *rmtC*, *sul1*
Plasmid 3	101,847	50.1	IncFII	*bla*_TEM-1_
Plasmid 4	4,145	50.8	NI^*a*^	
Plasmid 5	3,359	51.7	NI	
Plasmid 6	2,883	49.7	NI	
Plasmid 7	1,934	51.4	NI	
Plasmid 8	1,780	56.6	ColpVC	

a NI, not identified using PlasmidFinder v2.0.1.

### Data availability.

The complete genome sequences of E. coli NZRM4457 have been deposited in GenBank under accession numbers CP049967, CP049968, CP049969, CP049970, CP049971, CP049972, CP049973, CP049974, and CP049975 and under BioProject PRJNA611848. The raw reads were deposited into the NCBI Sequence Read Archive (SRA) under accession numbers SRR11292825 and SRR11300819.
